# Guideline-directed medical therapy is similarly effective in heart failure with mildly reduced ejection fraction

**DOI:** 10.1007/s00392-022-02053-8

**Published:** 2022-07-04

**Authors:** Sam Straw, Charlotte A. Cole, Melanie McGinlay, Michael Drozd, Thomas A. Slater, Judith E. Lowry, Maria F. Paton, Eylem Levelt, Richard M. Cubbon, Mark T. Kearney, Klaus K. Witte, John Gierula

**Affiliations:** 1 Leeds Institute of Cardiovascular and Metabolic Medicine, University of Leeds, Leeds, UK; 2 Leeds Teaching Hospitals NHS Trust, Leeds, UK; 3 Department of Internal Medicine I, University Clinic, RWTH Aachen University, Aachen, DE Germany

**Keywords:** Mildly reduced, Mid-range, Heart failure, Guideline-directed medical therapy

## Abstract

**Aims:**

Current guidelines recommend that disease-modifying pharmacological therapies may be considered for patients who have heart failure with mildly reduced ejection fraction (HFmrEF). We aimed to describe the characteristics, outcomes, provision of pharmacological therapies and dose-related associations with mortality risk in HFmrEF.

**Methods and results:**

We explored data from two prospective observational studies, which permitted the examination of the effects of pharmacological therapies across a broad spectrum of left ventricular ejection fraction (LVEF). The combined dataset consisted of 2388 unique patients, with a mean age of 73.7 ± 13.2 years of whom 1525 (63.9%) were male. LVEF ranged from 5 to 71% (mean 37.2 ± 12.8%) and 1504 (63.0%) were categorised as having reduced ejection fraction (HFrEF), 421 (17.6%) as HFmrEF and 463 (19.4%) as preserved ejection fraction (HFpEF). Patients with HFmrEF more closely resembled HFrEF than HFpEF. Adjusted all-cause mortality risk was lower in HFmrEF (hazard ratio [HR] 0.86 (95% confidence interval [CI] 0.74–0.99); *p* = 0.040) and in HFpEF (HR 0.61 (95% CI 0.52–0.71); *p* < 0.001) compared to HFrEF. Adjusted all-cause mortality risk was lower in patients with HFrEF and HFmrEF who received the highest doses of beta-blockers or renin-angiotensin inhibitors. These associations were not evident in HFpEF. Once adjusted for relevant confounders, each mg equivalent of bisoprolol (HR 0.95 [95% CI 0.91–1.00]; *p* = 0.047) and ramipril (HR 0.95 [95%CI 0.90–1.00]; *p* = 0.044) was associated with incremental reductions in mortality risk in patients with HFmrEF.

**Conclusions:**

Pharmacological therapies were associated with lower mortality risk in HFmrEF, supporting guideline recommendations which extend the indications of these agents to all patients with LVEF < 50%.

**Graphic abstract:**

HFmrEF more closely resembles HFrEF in terms of clinical characteristics and outcomes. Pharmacological therapies are associated with lower mortality risk in HFmrEF and HFrEF, but not in HFpEF.
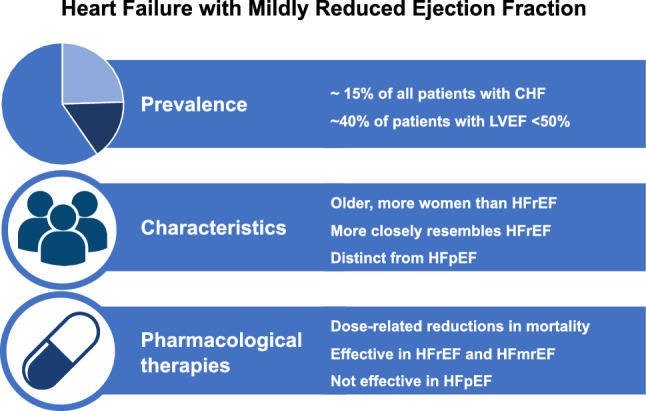

## Introduction

### Background

The benefits of disease-modifying pharmacological therapies for heart failure with reduced ejection fraction (HFrEF) are clear. Four classes of medications targeting the neurohormonal maladaptations of the syndrome are proven to reduce hospitalisations and improve survival [1]. Eligibility for these therapies is largely derived from the inclusion criteria of randomised controlled trials [2–6], which used arbitrary thresholds of left ventricular ejection fraction (LVEF) to identify patients perceived to be at the highest risk, who potentially had the most to gain. Current guidelines recommend that pharmacological therapies may be considered for people who have heart failure with mildly reduced ejection fraction (HFmrEF) (LVEF 41–49%) [7]. However, since this subgroup of patients was not included in the relevant trials, the benefits of these therapies are largely unknown, with these recommendations largely derived from consensus opinion, rather than published evidence. There is, therefore, a need to assess the impact of these recommendations in real-world populations.

We explored data from two prospective observational studies, which permitted the examination of the effects of pharmacological therapies across a broad spectrum of LVEF. Our aims were firstly, to report prevalence of HFrEF, HFmrEF and heart failure with preserved ejection fraction (HFpEF) amongst a real-world population referred to secondary care with symptoms of chronic heart failure (CHF). Secondly, to describe the clinical characteristics and outcomes of patients with HFmrEF compared to HFrEF and HFpEF, and thirdly, to report the provision of pharmacological therapies and explore dose-related associations with outcomes across heart failure classifications.

## Methods

### Study design

The United Kingdom Heart Failure Evaluation and Assessment of Risk Trial (UK-HEART-2) and the Prospective evaluation of the diagnostic efficacy of the 2010 United Kingdom National Institute for Clinical Excellence guidelines on Chronic Heart Failure (NICE-CHF) are two prospective, observational studies conducted within the same region in the UK. UK-HEART-2 represents a prevalent population of ambulatory patients under the care of four specialist heart failure outpatient clinics. Consecutive patients were approached to participate between July 2006 and December 2014. Inclusion required stable symptoms ± signs of CHF for at least 3 months and LVEF ≤ 45%. NICE-CHF represents a population of consecutive people newly referred to a specialist heart failure outpatient clinic, from a primary care catchment of over 750,000 people. We required patients to have symptoms ± signs of CHF and elevated natriuretic peptides (N-terminal pro B-type natriuretic peptide [NT-proBNP] ≥ 125 pg/L). All patients attending between May 2012 and May 2013 were included, regardless of LVEF.

Upon arrival at the outpatient heart failure clinic, demographic details, medical history, blood pressure, and for the UK-HEART-2 cohort functional capacity according to the New York Heart Association classification were recorded. A venous blood sample was taken at enrolment and tested for full blood count, creatinine, and albumin. For patients included in NICE-CHF, NT-proBNP was measured from samples taken in primary care using the Immulite 2000 assay (Siemens Healthcare Diagnostics, Camberley, UK) in the biochemistry laboratory at the Leeds Teaching Hospitals NHS Trust. The inter-batch coefficient of variation was 8.9% at 350 pg/mL and 5.9 at 4100 pg/mL. Standard 12-lead electrocardiograms were recorded at 25 mm/s and analysed by a senior cardiologist (RMC, MTK, KKW) blinded to patient characteristics. Two-dimensional transthoracic echocardiography was performed by senior cardiac sonographers (JG, MP, JEL), blinded to measurements of NT-proBNP. LV dimensions, LVEF, left atrial volumes and LV Doppler measurements were calculated according to the American Society of Echocardiography and European Association of Cardiovascular Imaging guidelines [8].

### Pharmacological therapies

Prescription of beta-blocker, angiotensin converting enzyme inhibitor (ACEi) or angiotensin receptor blocker (ARB) and loop diuretic were expressed as equivalent doses, relative to the maximum licensed dosages of bisoprolol, ramipril and furosemide as previously published [9]. For the purpose of analysis we divided the receipt of beta-blockers and ACEi/ARB into patients not receiving these agents, patients prescribed these at low doses (< 5 mg equivalent dose) and patients prescribed these agents at high doses (≥ 5 mg equivalent dose). In UK-HEART-2 medications were recorded at the time of study enrolment, in NICE-CHF, we recorded doses from linked primary care records. Both studies predated the availability of angiotensin receptor-neprilysin inhibitors (ARNI) and sodium-glucose co-transporter 2 inhibitors (SGLT2i).

### Patient classification and outcomes

Patients were categorised according to the Universal Definition and Classification of Heart Failure as having HFrEF, HFmrEF, HFpEF or not having CHF [10]. HFrEF and HFmrEF required symptoms ± signs of CHF and LVEF ≤ 40% and 41–49%, respectively. HFpEF required signs ± symptoms of CHF, elevated natriuretic peptides (NT-proBNP ≥ 125 pg/mL) as well as evidence of relevant structural heart disease (for example dilated left atrium or LV hypertrophy) or diastolic dysfunction. Patients without these features were regarded as not having CHF. Vital status data were collected using linked national electronic records from the Hospital Episode Statistics and Office of National Statistics. Final censorship occurred in November 2018 for UK-HEART-2 and April 2019 for NICE-CHF.

### Statistics

All statistical analyses were performed using IBM SPSS Statistics version 26 (IBM Corporation, Armonk, NY). Normality of distribution was explored visually by distribution plots and confirmed using skewness tests. Continuous variables are presented as mean ± standard deviation if normally distributed, as median (interquartile range) if non-normally distributed and discrete variables are presented as number (percentage). Groups were compared using two-sided t tests or one-way analysis of covariance for normally distributed continuous data, Mann–Whitey or Kruskal–Wallis H tests for non-normally distributed data, and two-sided Pearson *χ*^2^ for categorical variables. Kaplan–Meier analysis was used to plot survival and groups compared using log-rank test. Age-sex adjusted and multivariable analyses used Cox proportional hazards regression. In all analyses, statistical significance was defined as *p* < 0.05.

### Ethical considerations

The Health Research Authority provided ethical approval for the studies (UK-HEART-2: 07/Q1205/17; NICE-CHF: CAG8-03(PR1)/2013) which were conducted in accordance with the principles outlined in the Declaration of Helsinki. Participants enrolled in UK-HEART-2 provided informed written consent for inclusion. Ethical approval for NICE-CHF was achieved through a Sect. 251 application reviewed by the Confidential Advisory Group which allows individual patient data to be used for health service improvement without the need for individual patient consent.

## Results

### Classification of heart failure and distribution of ejection fraction

**Fig. 1:**
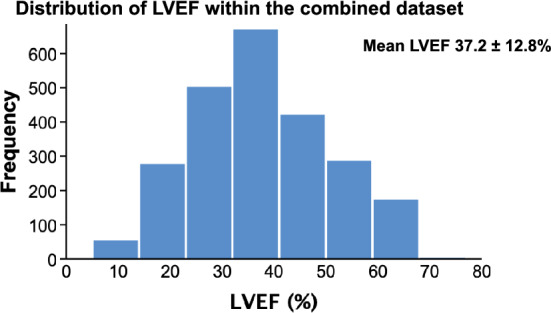
Histogram showing distribution of LVEF within the combined dataset.

UK-HEART-2 recruited a total of 1802 participants, 47 had insufficient endocardial definition to measure LVEF and five had missing medication doses, leaving 1750 patients, of whom 1423 (81.3%) were classified as having HFrEF and 327 (18.7%) as having HFmrEF. NICE-CHF included 982 patients, of these 22 had insufficient endocardial definition to measure LVEF, 182 did not have CHF and 2 had missing medication doses, leaving 776 patients, 190 (24.5%) of whom had HFrEF, 123 (15.9%) had HFmrEF and 463 (59.7%) had HFpEF. Following the exclusion of 138 duplicate entries for patients enrolled in both studies (due to inappropriate re-referral of patients enrolled in UK-HEART-2 through the NT-proBNP pathway for a new diagnosis of CHF), the combined dataset consisted of 2388 unique patients, who had a mean age of 73.7 ± 13.3 years and 1525 (63.9%) were male. Within the entire study cohort, LVEF ranged from 5 to 71% (mean 37.2 ± 12.8%) (Fig. 1). Overall, 1504 (63.0%) patients were categorised as having HFrEF, 421 (17.6%) as HFmrEF and 463 (19.4%) as HFpEF.

### Clinical characteristics

Descriptive data contrasting patients according to heart failure classification are displayed in Table 1. Patients with HFrEF had a lower mean age and were more likely to be male compared to HFmrEF, although distributions of ischaemic heart disease and diabetes mellitus were similar. Patients with HFpEF were more likely to be older and female, and fewer had a history of ischaemic heart disease. Aside from cardiac dysfunction, there was evidence of differing conventional markers of disease severity across the three classifications, with those with lower LVEF having more impaired renal function, higher NT-proBNP and lower blood pressure. Those with HFrEF were the most symptomatic, being more likely to have NYHA Class III/IV symptoms compared to HFmrEF (32.1% vs 25.1%; *p* = 0.013 in the UK-HEART-2 cohort) with higher mean dosing of loop diuretic in those with lower LVEF (*p* < 0.001 in the combined dataset).

**Table 1:** Clinical characteristics of patients according to classification of CHF

	All patients (n = 2388)	HFrEF (n = 1504)	HFmrEF (n = 421)	HFpEF (n = 463)
*Demographics*				
Age (years)	73.7 ± 13.2	70.4 ± 13.2	74.2 ± 12.4**	83.8 ± 8.6**
Male sex [*n* (%)]	1525 (63.9)	1099 (73.1)	266 (63.2)**	160 (34.6)**
NYHA Class III/IV^#^	539 (30.8)	457 (32.1)	82 (25.1)*	-
*Co-morbidities*				
IHD [*n* (%)]	1183 (49.5)	847 (56.3)	231 (54.9)	105 (22.7)**
Diabetes mellitus [*n* (%)]	660 (27.6)	414 (27.5)	134 (31.8)	112 (24.2)
COPD [*n* (%)]	364 (15.2)	226 (15.0)	74 (17.6)	64 (13.8)
*Observations*				
SBP (mmHg)	128.2 ± 24.0	122.1 ± 22.0	131.6 ± 22.4**	144.2 ± 23.4**
DBP (mmHg)	72.0 ± 11.8	71.2 ± 11.6	72.7 ± 12.1*	73.8 ± 12.0**
Heart rate (beats/min)	75.2 ± 17.6	76.0 ± 18.5	74.4 ± 17.0	73.5 ± 14.8*
*Echocardiogram*				
LVEDd (mm)	54.1 ± 9.8	58 (52.8–64)	50.0 ± 7.8**	44.9 ± 6.3**
LVEF (%)	37.2 ± 12.8	30 (24–36)	44.7 ± 1.8**	56.2 ± 4.0**
*Blood tests*				
Haemoglobin (g/L)	132.6 ± 19.4	134.6 ± 18.8	130.2 ± 20.5**	128.0 ± 19.2**
Creatinine (mmol/L)	102 (80–128)	107 (87–134)	102 (81–129.8)**	79 (66–102.5)**
Albumin (g/L)	42.5 ± 3.6	42.7 ± 3.6	42.6 ± 3.4	41.4 ± 3.5**
NT-proBNP (pg/mL)^#^	1054.5 (508.5–2555)	2511 (1009–5972)	1126 (511–2245)**	845 (438–1705)**
HbA1c (mmol/mol)^#^	45 (41–55)	46 (41–56)	51 (43–58)	44 (40–52.8)
*Medications*				
Beta-blocker [*n* (%)]	1875 (78.5)	1274 (84.7)	324 (77.0)**	277 (59.8)**
Bisoprolol dose (mg)	3.8 ± 3.4	4.0 ± 3.4	3.5 ± 3.4*	3.2 ± 3.6**
ACEi/ARB [*n* (%)]	1977 (82.8)	1332 (88.6)	356 (84.6)*	289 (62.4)**
Ramipril dose (mg)	4.5 ± 3.7	4.9 ± 3.6	4.4 ± 3.5*	3.4 ± 3.8**
Loop diuretic [*n* (%)]	1205 (66.5)	790 (75.3)	191 (63.7)**	224 (48.4)**
Furosemide dose (mg)	42.6 ± 46.7	50.2 ± 49.1	41.0 ± 46.4**	18.8 ± 25.9**
MRA [*n* (%)]	728 (30.5)	600 (39.9)	95 (22.6)**	33 (7.1)**

*IHD* ischaemic heart disease, *COPD* chronic obstructive pulmonary disease, *NYHA* New York Heart Association, *SBP* systolic blood pressure, *DBP* diastolic blood pressure, *LVEDd* left ventricular end-diastolic diameter, *NT-proBNP* N-terminal pro B-type natriuretic peptide, *HbA1c* glycosylated haemoglobin, *ACEi* angiotensin converting enzyme inhibitor, *ARB* angiotensin receptor blocker, *MRA* mineralocorticoid receptor antagonist

p* < 0.05, ** < 0.005 compared to HFrEF

^#^NYHA classification was not measured in NICE-CHF; NT-proBNP and HbA1c were not measured UK-HEART-2

### Provision of pharmacological therapies

Within the combined dataset, 1875 (78.5%) were prescribed a beta-blocker, 1977 (82.8%) an ACEi/ARB and 728 (30.5%) a mineralocorticoid receptor antagonist (MRA) (Table 1). Patients with HFrEF were the most likely to receive a beta-blocker (84.7%), ACEi/ARB (86.6%) or MRA (39.9%), whereas those with HFpEF were least likely (59.8%, 62.4% and 7.1%). Patients classified as having HFmrEF usually received a beta-blocker (77.0%) and ACEi/ARB (84.6%) but fewer received an MRA (22.6%). Mean dosing of beta-blockers and ACEi/ARB was different across the three classifications, with those with HFrEF prescribed the highest doses.

### Provision of pharmacological therapy and outcomes

**Fig. 2:**
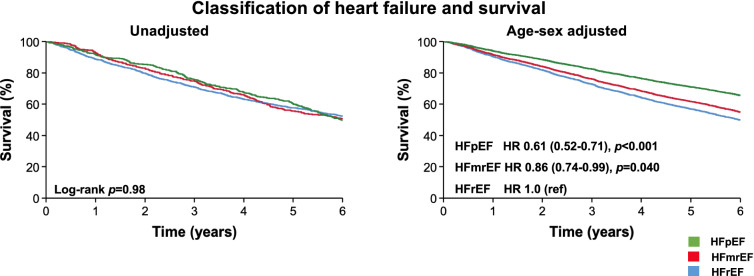
Kaplan–Meier and age-sex adjusted survival plots divided by classification of CHF.

**Fig. 3:**
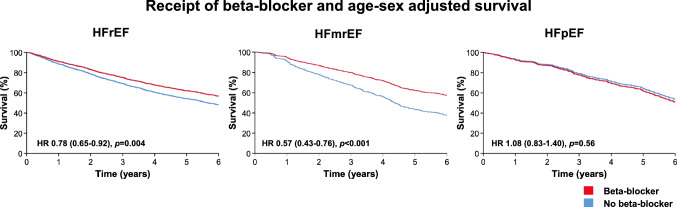
Age-sex adjusted survival plot according to receipt of beta-blocker divided by classification of CHF.

**Fig. 4:**
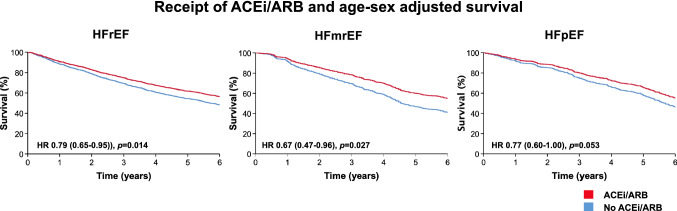
Age-sex adjusted survival plot according to receipt of ACEi/ARB divided by classification of CHF.

During a mean follow-up of 4.8 ± 2.1 years, a total of 1331 (55.7%) patients died. Unadjusted survival was not different between classifications of CHF (log-rank *p* = 0.98). However, in age-sex adjusted analysis, all-cause mortality risk was lower in HFmrEF (hazard ratio [HR] 0.86 (95% confidence interval [CI] 0.74–0.99); *p* = 0.040) and in HFpEF (HR 0.61 (95% CI 0.52–0.71); *p* < 0.001) than HFrEF (Fig. 2). Receipt of beta-blockers or ACEi/ARB was associated with better survival in all classifications of heart failure. In age-sex adjusted analysis, these associations remained evident for HFrEF and HFmrEF, but not for HFpEF (Figs. 3 and 4). The receipt of MRA was not associated with survival in HFrEF (*p* = 0.48) or HFmrEF (*p* = 0.74) but was associated with a worse prognosis in the small proportion of patients with HFpEF who received these agents (*p* = 0.001).

### Dosing of pharmacological therapies and outcomes

**Fig. 5:**
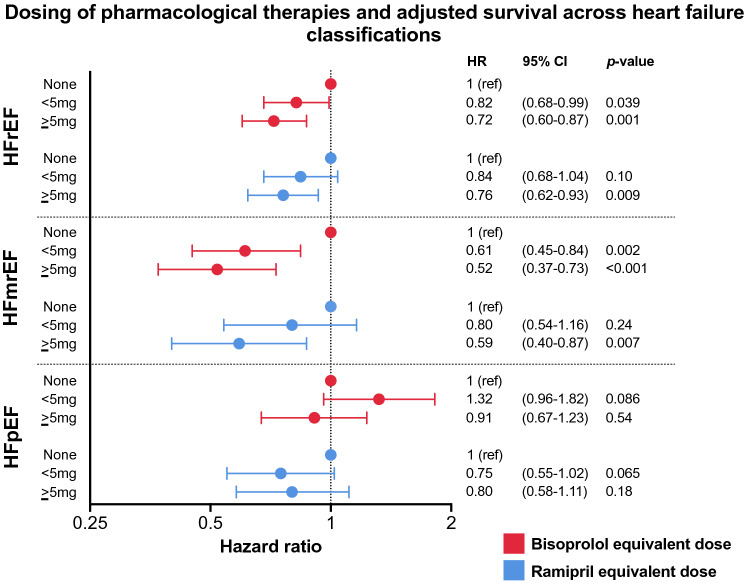
Forrest plot showing adjusted hazard ratio of all-cause mortality divided by dosing of beta-blocker and ACEi/ARB.

We explored the association of the receipt of pharmacological therapies and outcomes further by dividing patients with HFrEF, HFmrEF and HFpEF into those not receiving these agents, those prescribed low doses (< 5 mg equivalent dose) and those prescribed high doses (≥ 5 mg equivalent dose) of beta-blockers and ACEi/ARB. Higher dosing of beta-blockers and ACEi/ARBs was associated with lower all-cause mortality risk in patients with HFrEF and HFmrEF, but this was not the case in HFpEF (Fig. 5). There were also clear associations between beta-blocker and ACEi/ARB dosing group and patient characteristics, such as age, history of ischaemic heart disease and diabetes, and cardiac dysfunction, although the pattern of these association was similar between patients with HFrEF and HFmrEF (Tables 2 and 3).

**Table 2:** Clinical characteristics of patients with HFrEF and HFmrEF divided by dosing of beta-blocker

	HFrEF			HFmrEF		
	None (*n* = 229)	< 5 mg (*n* = 642)	≥ 5 mg (*n* = 631)	None (*n* = 95)	< 5 mg (*n* = 167)	≥ 5 mg (*n* = 156)
*Demographics*						
Age (years)	72.6 ± 12.6**	71.1 ± 13.1	68.8 ± 13.3	77.2 ± 12.0**	74.3 ± 11.1	71.8 ± 13.3
Male sex [*n* (%)]	157 (68.6)*	454 (70.7)	486 (77.0)	54 (56.8)*	112 (67.1)	98 (62.8)
*Co-morbidities*						
IHD [*n* (%)]	129 (56.3)	356 (55.5)	362 (57.4)	44 (46.3)	98 (58.7)	86 (55.1)
Diabetes mellitus [*n* (%)]	60 (26.2)*	153 (23.8)	200 (31.7)	24 (25.3)*	51 (30.5)	59 (37.8)
COPD [*n* (%)]	64 (27.9)**	104 (16.2)	56 (8.9)	30 (31.6)**	24 (14.4)	19 (12.2)
*Observations*						
SBP (mmHg)	124.4 ± 21.2	121.0 ± 22.2	122.1 ± 22.0	131.3 ± 22.5	132.4 ± 22.3	130.8 ± 22.7
DBP (mmHg)	71.9 ± 11.3	70.6 ± 11.3	71.6 ± 11.8	73.1 ± 12.3	72.5 ± 12.4	72.6 ± 11.9
Heart rate (beats/min)	79.2 ± 18.9*	75.3 ± 18.3	75.5 ± 18.6	76.2 ± 15.7	73.1 ± 15.6	74.7 ± 19.1
*Echocardiogram*						
LVEDd (mm)	57.0 ± 9.3	58.3 ± 8.8	58.6 ± 8.3	47.5 ± 8.5**	50.4 ± 7.5	51.2 ± 7.3
LVEF (%)	30.8 ± 7.8**	28.7 ± 8.5	29.4 ± 8.0	45.1 ± 2.1**	44.3 ± 1.6	44.8 ± 1.7
*Blood tests*						
Haemoglobin (g/L)	132.3 ± 19.4*	133.9 ± 18.7	134.6 ± 18.7	131.7 ± 17.5	129.1 ± 20.8	131.4 ± 20.1
Creatinine (mmol/L)	107 (83.3–135.8)	106 (86–133)	108 (90.8–133.3)	99 (77–124)	107 (83–135)	99 (80–122)
Albumin (g/L)	41.9 ± 3.7**	42.6 ± 3.9	43.2 ± 3.3	42.5 ± 3.7	42.3 ± 3.5	43.1 ± 2.9

*IHD* ischaemic heart disease, *COPD* chronic obstructive pulmonary disease, *NYHA* New York Heart Association, *SBP* systolic blood pressure, *DBP* diastolic blood pressure, *LVEDd* left ventricular end-diastolic diameter, *NT-proBNP* N-terminal pro B-type natriuretic peptide, *HbA1c* glycosylated haemoglobin, *ACEi* angiotensin converting enzyme inhibitor, *ARB* angiotensin receptor blocker, *MRA* mineralocorticoid receptor antagonist

*p < 0.05, **p < 0.005 across three groups

**Table 3:** Clinical characteristics of patients with HFrEF and HFmrEF divided by dosing of ACEi/ARB

	HFrEF			HFmrEF		
	None (*n* = 169)	< 5 mg (*n* = 514)	≥ 5 mg (*n* = 819)	None (*n* = 65)	< 5 mg (*n* = 148)	≥ 5 mg (*n* = 206)
*Demographics*						
Age (years)	75.6 ± 11.7**	70.8 ± 14.0	69.0 ± 12.7	79.5 ± 11.7**	74.3 ± 12.7	72.3 ± 11.9
Male sex [*n* (%)]	110 (65.1)**	358 (69.6)	630 (76.9)	30 (46.2)**	86 (58.1)	149 (72.3)
*Co-morbidities*						
IHD [*n* (%)]	102 (60.4)	277 (53.9)	467 (57.0)	27 (41.5)*	80 (54.1)	124 (60.2)
Diabetes mellitus [*n *(%)]	45 (26.6)	125 (24.3)	244 (29.8)	19 (29.2)	47 (31.8)	67 (32.5)
COPD [*n* (%)]	26 (15.4)	81 (15.8)	118 (14.4)	15 (23.1)	31 (20.9)	28 (13.6)
*Observations*						
SBP (mmHg)	125.1 ± 21.7*	119.6 ± 22.6	123.0 ± 21.5	131.5 ± 21.4	129.8 ± 21.8	133.1 ± 23.2
DBP (mmHg)	71.5 ± 12.1*	70.0 ± 11.1	71.9 ± 11.7	71.4 ± 11.6	72.3 ± 11.9	73.5 ± 12.6
Heart rate (beats/min)	78.2 ± 19.6*	77.3 ± 19.1	74.6 ± 17.8	78.3 ± 18.0*	76.4 ± 17.7	71.8 ± 15.9
*Echocardiogram*						
LVEDd (mm)	55.7 ± 9.2**	57.9 ± 8.7	58.9 ± 8.5	46.7 ± 7.1**	49.6 ± 8.5	51.3 ± 7.1
LVEF (%)	30.5 ± 7.6	29.0 ± 8.5	29.3 ± 8.4	45.4 ± 2.1**	44.6 ± 1.8	44.5 ± 1.7
*Blood tests*						
Haemoglobin (g/L)	129.1 ± 18.5**	133.8 ± 18.6	136.2 ± 18.8	124.9 ± 22.5	130.8 ± 18.5	131.4 ± 21.2
Creatinine (mmol/L)	117 (94–167)**	106 (85–134)	106 (87–131)	91 (72.5–140)	98 (78.3–128.8)	105 (86.5–130.5)
Albumin (g/L)	41.5 ± 3.8**	42.3 ± 3.6	43.3 ± 3.5	41.1 ± 3.5**	42.4 ± 3.5	43.3 ± 3.1

*IHD* ischaemic heart disease, *COPD* chronic obstructive pulmonary disease, *NYHA* New York Heart Association, *SBP* systolic blood pressure, *DBP* diastolic blood pressure, *LVEDd* left ventricular end-diastolic diameter, *NT-proBNP* N-terminal pro B-type natriuretic peptide, *HbA1c* glycosylated haemoglobin, *ACEi* angiotensin converting enzyme inhibitor, *ARB* angiotensin receptor blocker, *MRA* mineralocorticoid receptor antagonist

*p < 0.05, **p < 0.005 across three groups

We then used Cox regression to further define the association between dosing of beta-blockers and ACEi/ARB and all-cause mortality risk in HFrEF and HFmrEF. Interaction analyses suggested that LVEF (as a continuous variable) was not a significant modifier of the effect of the dosing of beta-blockers (*p* = 0.83) or ACEi/ARB (*p* = 0.91) in patients with LVEF < 50%. Regression models including factors associated with dosing of beta-blockers and ACEi/ARB were used to determine the association of dosing of these agents with all-cause mortality risk. In a model including age, sex, diabetes mellitus, chronic obstructive pulmonary disease, heart rate, LVEF, serum haemoglobin, and albumin (which were all associated with dosing of beta-blocker), each mg equivalent dose of bisoprolol was associated with incremental reductions in all-cause mortality risk in HFmrEF (HR 0.95 (95% CI 0.91–1.00); *p* = 0.047). Similarly, when adjusted for age, sex, systolic and diastolic blood pressure, heart rate, serum haemoglobin, creatinine and albumin (which were all associated with dosing of ACEi/ARB) each mg equivalent of ramipril was associated with a similar magnitude of reduction in all-cause mortality risk (HR 0.95 (95% CI 0.90–1.0); *p* = 0.044).

## Discussion

In this pooled analysis of two prospective observational studies, we examined the provision of pharmacological therapies and dosing-related associations with mortality risk across a broad spectrum of LVEF. We were able to show that: (1) HFmrEF is highly prevalent amongst patients presenting to secondary care with symptoms of CHF and elevated natriuretic peptides; (2) clinical characteristics and outcomes varied according to LVEF, but patients with HFmrEF more closely resembled HFrEF, than HFpEF; and (3) higher dosing of beta-blockers and ACEi/ARB was associated with better survival in HFrEF and HFmrEF, but not in HFpEF. Taken together, our findings support guideline recommendations extending the indications of pharmacological therapies to all patients with CHF and LV systolic dysfunction.

### Prevalence and characteristics of heart failure with mildly reduced ejection fraction

Although the combined dataset included patients enrolled in UK-HEART-2 which excluded people with LVEF > 45%, by separately reporting data from the NICE-CHF study, we were able to show that in an unselected cohort referred to secondary care with symptoms ± signs of CHF and elevated natriuretic peptides [11], ~ 75% had LVEF > 40%. HFmrEF and HFpEF therefore represent highly prevalent populations, for which therapeutic strategies are required. Our findings that a substantial proportion of patients encountered in clinical practice have HFmrEF, are consistent with other registry studies. For example, the prevalence of HFmrEF in the Swedish Heart Failure Registry was around ~ 25%, although the proportion was only ~ 8% in the Get with the Guidelines Heart Failure (GWTG-HF) registry [12].

We observed differences in the baseline characteristics of patients with HFmrEF compared to HFrEF. However, although these differences were statistically significant, the numerical differences between groups were relatively small, and the distribution of co-morbidities was similar. Overall, the clinical characteristics of patients with HFmrEF more closely resembled those of HFrEF. The baseline characteristics of patients with HFpEF were more distinct. HFpEF patients were more likely to be older, to be female and had biomarker evidence of less severe clinical heart failure, for example, lower natriuretic peptides, higher blood pressure and better renal function.

Prior registry and interventional studies have reached conflicting conclusions as to whether patients with HFmrEF more closely resemble HFrEF, HFpEF or are a group with distinct clinical characteristics and outcomes. Earlier studies, for example the GWTG-HF registry, suggest that these patients were more similar to those with HFpEF, and furthermore, showed no difference in adjusted survival rates across the heart failure classifications [12]. In comparison, in the Candesartan in Heart Failure—Assessment of Reduction in Mortality and Morbidity (CHARM) programme, whilst those with HFrEF and HFmrEF were similar in terms of age, sex distribution and history of myocardial infarction, those with HFmrEF had a lower risk of cardiovascular death or hospitalisation for heart failure [13]. The Chronic Heart Failure Analysis and Registry in Tohoku District-2 (CHART-2) study suggested that HFmrEF represents an intermediate risk group on the continuity of LVEF, and furthermore made the observation that patients often transitioned to between these groups—especially from HFrEF or HFmrEF to HFpEF due to LV reverse remodelling [14]. This approach seems biologically the most plausible. The lack of longitudinal imaging data means that the current study was unable to assess outcomes for patients with heart failure with improved ejection fraction, although it is generally accepted that such patients continue to derive benefit from pharmacological therapies [15].

### Heart failure classification and outcomes

Consistent with other reports [16], unadjusted survival was not different between classifications of heart failure. However, there were significant differences in mean ages and the distribution of sex across heart failure classifications. Compared to HFrEF, in age-sex adjusted analyses we observed better survival in HFpEF (HR 0.61 [95% CI 0.52–0.71]) and marginally better survival in HFmrEF (HR 0.86 [95% CI 0.74–0.99]). Across all three classifications of heart failure, we observed better survival in unadjusted analyses with the receipt of pharmacological therapies. However, once adjusted for age and sex, these associations were no longer evident in HFpEF.

For the first time, guidelines recommend that pharmacological therapies approved for HFrEF may be considered for those with LVEF 41–49% [7], supporting what may have been routine practice in many settings for some time. In the absence of randomised trials, our approach was to administer beta-blockers and ACEi/ARB for those presenting with signs and symptoms of CHF and LVEF < 50% who were able to tolerate these agents. Consequently, most patients attending our service who had LVEF 41–49% received these agents. Receipt of higher doses of beta-blockers and ACEi/ARB was associated with a lower all-cause mortality risk, even after adjusting for confounding variables. These observations suggest benefit in patients with less severely impaired cardiac function, which allies well the hypothesis that heart failure progression can be slowed, halted or even reversed more effectively during its early stages. On the other hand, consistent with the lack of clinical trial evidence for HFpEF [17, 18] treatments offered to this group attending our service were limited to lifestyle and risk factor modification as well as loop diuretics for alleviation of symptoms of congestion. Hence, the use of disease-modifying agents in those with HFpEF was lower, and we did not observe better outcomes in those receiving these agents.

### Pharmacological therapies and outcomes for heart failure with mildly reduced ejection fraction

There are no cardiovascular outcomes trials specifically designed to evaluate the efficacy of pharmacological therapies in HFmrEF [19]. Furthermore, patients with HFmrEF have traditionally been excluded from trials in HFrEF. On the other hand, many studies investigating outcomes in HFpEF (especially more recent trials) have had inclusion criteria overlapping the 41–49% LVEF range, providing insights into the management of patients who would be classified as having HFmrEF according to the proposed universal definition [10]. The CHARM programme consisted of three clinical trials evaluating the effects of candesartan. CHARM-Preserved (LVEF > 40%) did not demonstrate reductions in mortality with candesartan [20]. However, in a pooled analysis, there were reductions in the primary end-point of cardiovascular death or hospitalisation for heart failure in those with LVEF 40–49% (HR 0.76 [95% CI 0.61–0.96]; *p* = 0.02) [21]. Similarly, the Prospective Comparison of ARNI with ARB Global Outcomes in Heart Failure with Preserved Ejection Fraction (PARAGON-HF) trial assessing the efficacy of sacubitril-valsartan in participants with LVEF ≥ 45%, did not demonstrate improved outcomes with the novel agent. However, when pooled with the Prospective Comparison of ARNI with ACEI to Determine Impact on Global Mortality and Morbidity in Heart Failure (PARADIGM-HF) trial (LVEF ≤ 40%), although the therapeutic effects of sacubitril-valsartan were found to be greatest in those with the most marked LV systolic dysfunction, the benefits did extend to those classified as having HFmrEF [22]. Finally, in the Treatment of Preserved Cardiac Function Heart Failure with an Aldosterone Antagonist (TOPCAT) trial (LVEF ≥ 45%) [23], patient characteristics and the treatment effect of spironolactone were substantially modified by LVEF, with the greatest benefit (although non-significant) amongst those with LVEF < 50% (HR 0.72 [95% CI 0.50–1.05]) [24].

For all patients with HFrEF, guidelines recommend four classes of medications proven to reduce hospitalisations and cardiovascular mortality [25]. Adherence to guideline recommendations is associated with improved outcomes in real-world populations [26–28]. However, whether these recommendations for patients with HFmrEF will be translated into meaningful improvements in outcomes is less certain, given that the underpinning data are derived from clinical trials in which the overall results were neutral.

Recent observational studies also lend support to the notion that pharmacological therapies may improve outcomes in HFmrEF. In the Swedish Heart Failure Registry, the provision of an ACEi/ARB was associated with lower all-cause mortality. However, the association for beta-blockers was only evident in those with coronary artery disease [29]. The CHART-2 registry showed associations with lower mortality with the receipt of beta-blockers, but not ACEi/ARB [14]. On the other hand, in the Organized Program to Initiate Lifesaving Treatment in Hospitalized Patients with Heart Failure (OPTIMIZE-HF) registry beta-blockers were not associated with reductions in all-cause mortality in those with LVEF ≥ 40% [30]. Additionally, although recent studies have reported dosing of pharmacological therapies in real-world populations with CHF, these have typically excluded those with LVEF > 40% [26, 31, 32], or have not specifically reported outcomes for this population [33]. By separately reporting the provision of pharmacological therapies and outcomes for patients with HFmrEF, we were able to report the novel observation of incremental reductions in mortality risk amongst patients receiving the highest doses of these agents, plausibly supporting their efficacy in this setting.

### Strengths and limitations

Our analysis included patients from two prospective studies representative of real-world populations of patients with CHF, encompassing a broad spectrum of LVEF and categorised according to guideline recommendations [10]. Some limitations should be noted. This was an analysis of observational, non-randomised studies, our data are, therefore, susceptible to measured and unmeasured confounders. Medications were prescribed at the discretion of the treating cardiologist. The lack of randomisation means causality cannot be inferred and our findings should be regarded as hypothesis generating. Although our intention was to prescribe ACEi/ARB and beta-blockers for patients with HFmrEF who could tolerate these agents, the lack of standard operating procedures means that these data may be susceptible to indication bias. Fewer patients who had HFmrEF received these agents compared to HFrEF, although the differences in mean dosing of ACEi/ARB and beta-blockers were small suggesting that once the decision to initiate therapy has been made, titration is generally successful. Although UK-HEART-2 recruited from four centres in the UK, NICE-CHF data originate from one service which may limit generalisability, although the diverse characteristics of the area served by our centre mitigates against this [34]. Both studies predate the availability of ARNI and SGLT2i, which may be similarly effective in HFmrEF [35, 36] and the lack of longitudinal cardiac imaging data means these our findings may not be generalisable to those with an improved ejection fraction.

## Conclusion

Patients classified as having HFmrEF according to the Universal Definition and Classification of Heart Failure seem to derive dose-dependent benefits from pharmacological therapies on a par with patients with HFrEF. These findings lend support to guideline recommendations which extend the indications of pharmacological therapies to all patients with symptoms of CHF and LV systolic dysfunction.

## Data Availability

The datasets generated and/or analysed during the current study are not publicly available due to the inclusion of potentially identifiable information but are available from the corresponding author upon reasonable request.

## Abbreviations

HFrEF: Heart failure with reduced ejection fraction
LVEF: Left ventricular ejection fraction
HFmrEF: Heart failure with mildly reduced ejection fraction
HFpEF: Heart failure with preserved ejection fraction
CHF: Chronic heart failure
UK-HEART-2: United Kingdom heart failure evaluation and assessment of risk trial
NICE-CHF: Prospective evaluation of the diagnostic efficacy of the 2010 United Kingdom National Institute for Clinical Excellence Guidelines on Chronic Heart Failure
NT-proBNP: N-terminal pro B-type natriuretic peptide
ACEi: Angiotensin converting enzyme inhibitor
ARB: Angiotensin receptor blocker
ARNI: Angiotensin receptor-neprilysin inhibitors (ARNI)
SGLT2i: Sodium-glucose co-transporter 2 inhibitors
HR: Hazard ratio
CI: Confidence interval
GWTG-HF: Get with the Guidelines Heart Failure
CHARM: Candesartan in Heart Failure—Assessment of Reduction in Mortality and Morbidity
CHART-2: Chronic heart failure analysis and registry in Tohoku district-2
PARAGON-HF: Prospective comparison of ARNI with ARB global outcomes in heart failure with preserved ejection fraction (PARAGON-HF)
PARADIGM-HF: Prospective comparison of ARNI with ACEI to determine impact on global mortality and morbidity in heart failure
TOPCAT: Treatment of preserved cardiac function heart failure with an aldosterone antagonist
OPTIMIZE-HF: Organized program to initiate lifesaving treatment in hospitalized patients with heart failure
